# Altered static and dynamic functional connectivity of habenula in first-episode, drug-naïve schizophrenia patients, and their association with symptoms including hallucination and anxiety

**DOI:** 10.3389/fpsyt.2023.1078779

**Published:** 2023-01-19

**Authors:** Kangkang Xue, Jingli Chen, Yarui Wei, Yuan Chen, Shaoqiang Han, Caihong Wang, Yong Zhang, Xueqin Song, Jingliang Cheng

**Affiliations:** ^1^Department of Magnetic Resonance Imaging, The First Affiliated Hospital of Zhengzhou University, Zhengzhou, China; ^2^Department of Psychiatry, The First Affiliated Hospital of Zhengzhou University, Zhengzhou, China

**Keywords:** schizophrenia, habenula, static functional connectivity, dynamic functional connectivity, hallucination, anxiety

## Abstract

**Background and objective:**

The pathogenesis of schizophrenia (SCH) is related to the dysfunction of monoamine neurotransmitters, and the habenula participates in regulating the synthesis and release of dopamine. We examined the static functional connectivity (sFC) and dynamic functional connectivity (dFC) of habenula in first-episode schizophrenia patients using resting state functional magnetic resonance imaging (rs-fMRI) in this study.

**Methods:**

A total of 198 first-Episode, drug-Naïve schizophrenia patients and 199 healthy controls (HC) underwent rs-fMRI examinations. The sFC and dFC analysis with habenula as seed was performed to produce a whole-brain diagram initially, which subsequently were compared between SCH and HC groups. Finally, the correlation analysis of sFC and dFC values with the Positive and Negative Symptom Scale (PANSS) were performed.

**Results:**

Compared with the HC groups, the left habenula showed increased sFC with the bilateral middle temporal gyrus, bilateral superior temporal gyrus, and right temporal pole in the SCH group, and the right habenula exhibited increased sFC with the left middle temporal gyrus, left superior temporal gyrus, and left angular gyrus. Additionally, compared with the HC group, the left habenula showed decreased dFC with the bilateral cuneus gyrus and bilateral calcarine gyrus in the SCH group. The PANSS negative sub-scores were positively correlated with the sFC values of the bilateral habenula with the bilateral middle temporal gyrus, superior temporal gyrus and angular gyrus. The PANSS general sub-scores were positively correlated with the sFC values of the right habenula with the left middle temporal gyrus and left superior temporal gyrus. The hallucination scores of PANSS were negatively correlated with the sFC values of the left habenula with the bilateral cuneus gyrus and bilateral calcarine gyrus; The anxiety scores of PANSS were positively correlated with the dFC values of the left habenula with the right temporal pole.

**Conclusion:**

This study provides evidence that the habenula of the first-episode schizophrenia patients presented abnormal static functional connectivity with temporal lobe and angular gyrus, and additionally showed weakened stability of functional connectivity in occipital lobe. This abnormality is closely related to the symptoms of hallucination and anxiety in schizophrenia, which may indicate that the habenula involved in the pathophysiology of schizophrenia by affecting the dopamine pathway.

## 1. Introduction

Schizophrenia is a psychiatric disorder caused by brain dysfunction under the influence of biological, psychological and social environmental factors, which lead to different levels of obstacles in cognitive, emotional, volition and behavior and other mental activities, seriously threatening human health and quality of life, and causing huge burden to families and society (1). The clinical symptoms of schizophrenia are extremely knotty and vary greatly among individuals, including positive symptoms (hallucinations, delusions and mental disorders), negative symptoms (apathy, poverty of thought and spontaneous speech) and cognitive dysfunction (2).

Schizophrenia is a psychiatric disorder of unknown etiology. Researchers have been exposing the pathogenesis of schizophrenia from many aspects including genetics, neuroanatomy, neurobiochemistry, neurodevelopment, neuropathology, neuropsychology and so on. In neurobiochemical researches of schizophrenia, dopamine has recently garnered the most attention among the monoamine neurotransmitters, as studies in the past have demonstrated that its malfunction plays a significant part in the development of schizophrenia (3, 4). Dopamine is one of the important neurotransmitters that transmit information between brain neurons. It is produced by the ventral tegmentum and substantia nigra of the midbrain and can act on the prefrontal lobe, nucleus accumbens and striatum. It is believed that the dysfunction of dopaminergic neurotransmitter leads to the appearance of symptoms of schizophrenia (5). Patients with schizophrenia typically experience abnormalities in the frontal cortex’s dopamine D1 receptor hypofunction and the subcortical structure’s dopamine D2 receptor hyperfunction. Three crucial components in the pathogenesis of schizophrenia involving the prefrontal cortex, the limbic system, and the ventral tegmentum of the midbrain form three important neural circuits, namely, midbrain-limbic dopamine circuit, midbrain-cortex dopamine circuit and cortex-limbic glutamate circuit-γ-aminobutyric acid circuit (6). Whether the antipsychotic drugs are effective for schizophrenia is closely related to their affinity with dopamine D2 receptors, and researchers have widely accepted the “hyper dopamine hypothesis” according to extensive research bases. According to previous research, dopamine hypofunction in the prefrontal lobe is linked to negative symptoms like apathy and cognitive decline while dopamine hyperfunction in subcortical areas is linked to positive symptoms like hallucinations and delusions (7). Therefore, considering the key influence of dopamine dysfunction in the development of schizophrenia, to comprehend the pathophysiology of schizophrenia better, it is crucial to investigate the brain regions that control the midbrain dopamine system.

The habenula is a pair of small nuclei in the middle and posterior part of the dorsal thalamus, which is segmented into medial habenula (MHb) and lateral habenula (LHb) histologically. It is a relay station connecting the limbic system, the frontal lobe, the basal ganglia and the monoaminergic nuclei in the midbrain, relating to learning, reproduction, stress and anxiety reactions, pain, and reward (8). Studies have shown that dysfunction of habenula can lead to a variety of mental diseases, such as schizophrenia and depression (9, 10). The habenula, as an important node in the dopamine reward circuit and its role in mental diseases, as well as its biological function in regulating dopamine and 5-hydroxytryptamine (5-HT), has attracted extensive attention in recent years. The habenula gradually emerged as a new hotspot in the field of psychiatric research. In February, Cui et al. and Yang et al. published two research articles in Nature, which revealed the close relationship between depression and habenula, and was regarded as a major breakthrough in the study of mental diseases (11, 12). In terms of schizophrenia, numerous studies on animal have found that the functional disturbance of habenula is closely related to schizophrenia, but few studies of habenula in schizophrenia patients have been reported. A research on rats found that the decrease of attention, cognitive ability and social behavior activity after LHb damage was very similar to the symptoms of schizophrenia, and distraction could also cause the decline of learning and memory ability of rats, indicating that poor habenula function may act a crucial part in cognitive dysfunction of schizophrenia (13). Shepard et al. (9) believed that habenula dysfunction would limit the ability to learn from mistakes, which is one of the most typical cognitive deficits in schizophrenia. Studies on schizophrenia patients have found that the decrease in the volume, frequent calcification and hypometabolic state of habenula imply that the activity of habenula may be diminished in schizophrenia, which may cause the emergence of schizophrenia-like symptoms through increasing the midbrain dopaminergic activity (14).

Functional magnetic resonance imaging (fMRI) can be employed to detect resting and task states changes of brain regions in schizophrenia patients. Several studies have confirmed that schizophrenia patients show abnormal functional activities of brain regions at rest (15, 16). Functional connectivity is a basic indicator of brain network changes in patients with mental disorders, which can reveal additional information regarding brain function. So far, most functional connectivity (FC) studies on schizophrenia focus on static functional connectivity (sFC), and it reflects the statistical correlation of time series among various areas and supposes that the FC between regions is static across time (17, 18). In recent years, progressively more scholars believe that FC in resting state is dynamic, showing significant spontaneous fluctuations over a small time scale (19–23). Dynamic functional connectivity (dFC), a supplementary technique, can describe the dynamic properties of blood oxygenation level-dependent (BOLD) signal variations in the time scale. A fixed length time window is chosen for the dFC method and used to calculate FC metrics. After a predetermined duration, the window slides to the next time window, resulting in several FC metrics that can clarify the time characteristics of FC over the entire scan duration. The dFC method can accurately describe the cooperation of brain regions by calculating the time-varying covariance of bold signals between regions. In other words, the higher the dFC value, the more unstable the FC. The instability of FC may reveal the neural mechanism of diverse diseases, numerous studies have reported that the FC window states of healthy controls were observed to switch more frequently, indicating that patients with disorders such as schizophrenia and depression tend to linger in a state of “weak” and relatively “rigid” connectivity with a decreased dFC, while healthy controls dynamically switch between different FC states. Such a finding was not detectable using a static FC approach, and underscores the importance of evaluating dynamic changes in connectivity. Yet the relationship between more unstable FC with better or worse symptomatology still need further discussion. Therefore, the functional connectivity between different brain regions changes with time, and dFC reflects the variation of functional connectivity on the time scale, which can explain the stability of brain activity more deeply and contribute to clarify the pathogenesis of schizophrenia. Consequently, combining sFC and dFC methods to analyze the brain functional images of schizophrenia patients can better explain its neural mechanisms. However, there are few studies on habenula activity in schizophrenia patients. Shepard et al. found that the habenula regulates dopamine neurons and negative symptoms in patients with schizophrenia in their study (9) and Zhang et al. also found that schizophrenia patients presented abnormal volume and functional connectivity compared with healthy controls (24). However, none of these studies has discovered the dynamic functional connectivity of the habenula. So, it is unclear whether and how the habenula function is altered in schizophrenia patients, and there is no study on dFC of habenula in schizophrenia patients.

The current study’s objective was to explore the sFC and dFC of habenula in schizophrenia patients, comprehensively explore the role of habenula in the pathophysiological changes of schizophrenia, search for the biological markers of the disease from the perspective of brain function, and provide clues and basis for the clinical diagnosis and evaluation of schizophrenia. This study collected rs-fMRI data from first-episode and drug- naïve schizophrenia patients and healthy controls. First, we studied the voxel-wise sFC and dFC of bilateral habenula with the whole brain in two groups; Additionally, we conducted the correlation analysis of altered sFC and dFC values with symptom index in schizophrenia (SCH) group. We hypothesized that SCH may show altered sFC and dynamic FC fluctuations of habenula throughout the scanning compared with the healthy controls (HC) group, which made it possible to substantially discover the neuromechanism of schizophrenia.

## 2. Materials and methods

### 2.1. Participants

We recruited 198 first-episode drug-naïve schizophrenia patients and 199 healthy controls, and all participants are Chinese Han and right-handed. According to the Diagnostic and Statistical Manual of Mental Disorders, Fourth Edition (DSM-IV), schizophrenia was diagnosed by two professional clinical psychiatrists. The schizophrenia patients included in this study were recruited from the psychiatry department of the First Affiliated Hospital of Zhengzhou University, and never received treatment and psychological counseling before, with the first symptom of which (disease’s duration) occurs less than 3 years. According to the DSM-IV diagnostic criteria and the definition of “first episode” by Breitborde et al. and Keshavan and Schooler (25, 26), it is reasonable to infer that drug-naïve patients with a course of less than 3 years can be regarded as a group of first-episode schizophrenia included in this study, yet the possibility cannot be ruled out that some of the included patients were not first episode. Two trained researchers assessed the patients with questionnaires and assessed their symptoms with the Positive and Negative Symptom Scales (PANSS).

The present study was approved by the Ethics Committee of the First Affiliated Hospital of Zhengzhou University. The exclusion criteria for this study are as follows: (1) The head has suffered from trauma or severe organic disease; (2) Drug or alcohol abuse; (3) Organic mental disease; (4) Pregnancy; and (5) MRI contraindication. For HC group, nervous system disease or mental illness, as well as family history of mental illness will be excluded. After clarifying the study, all participants were asked to sign the informed consent sheet.

### 2.2. Data acquisition

Magnetic resonance imaging (MRI) data were acquired by 3.0 T MRI scanner (Discovery MR750, GE, USA) with an 8-channel head coil. All subjects were asked to lie quietly with their eyes closed, stay awake and relaxed, foam pads and rubber earplugs being used to reduce head movement and strepitous interference.

A 3D-T1 BRAVO sequence was applied to obtain high-resolution structural images with the following parameters: repetition time (TR)/echo time (TE) = 8.2/3.2 ms, slices = 188, slice thickness = 1 mm, slice gap = 0 mm, flip angle (FA) = 12°, field of view (FOV) = 25.6 × 25.6 cm^2^, number of averages = 1, data matrix = 256 × 256, voxel size = 1 × 1 × 1 mm^3^, scan time = 4.33 min. The functional images were obtained using a gradient spin echo planar imaging (EPI) sequence: TR/TE = 2000/30 ms, slices = 32, slice thickness = 4 mm, slice gap = 0.5 mm, FA = 90°, field of view (FOV) = 22 × 22 cm^2^, number of averages = 1, data matrix = 64 × 64, voxel size = 3.4375 × 3.4375 × 4 mm^3^, and 180 volumes lasting for 360 s.

### 2.3. Data preprocessing

Based on MATLAB (MathWorks) platform, DPABI toolbox^1^ was used to preprocess rs-fMRI data. To allow magnetic saturation, the first five volumes of data were thrown away. Next, additional preprocessing was conducted with the following procedures: (1) slice timing; (2) realignment, subjects with maximum head motion exceeding 3 mm or a 3° rotation will be removed for purpose of head motion rectification; (3) normalization (DARTEL method), the unified structural images segmentation information were used to register individual functional images to the Montreal Neurological Institute (MNI) coordinate space, resampling to 3 × 3 × 3 mm^3^; (4) detrending; (5) temporal band-pass filtering (0.01–0.08 Hz); and (6) nuisance covariates regression of white matter, cerebrospinal fluid and head movement profile (Friston-24).

### 2.4. Definition of region of interest

Two regions of interest (ROIs) were selected to conduct sFC and dFC analysis, including left habenula and right habenula. The bilateral habenula mask was downloaded on the website of Automated Human Habenula Segmentation Program^2^ according to a previously calculated functional magnetic resonance imaging study (27).

### 2.5. Static FC analyses

The Data Processing Assistant for Resting-State fMRI Advanced Edition (DPARSFA) software package in DPABI software is used for sFC analysis. The BOLD time series of the left and right habenula was extracted respectively, then the Pearson correlation coefficients with the whole brain’s time series were measured, and finally the Fisher Z transformation values were used for subsequent statistical analysis.

### 2.6. Dynamic FC analyses

The Temporary Dynamic Analysis software package in DPABI software is used for dFC analysis with the sliding window method (28), which was employed to form dFC maps for each subject. According to the recommendations on dFC in previous researches (29), in order to optimize the balance between the characteristics of rapid functional status changes and the stable connectivity among brain regions, the appropriate length for obtaining dynamic FC state is 30 seconds to 1 minute using the Hamming window method (20). Finally, the window length is set to 30 TR (60s) and the sliding step length is 1 TR. In each window, Pearson correlation coefficients of the whole brain’s time series were measured were calculated, generating FC maps of left and right habenula of each window, and then Fisher Z transformation was performed to generate zFC maps. The standard deviation of zFC value of each window is the dFC value.

### 2.7. Statistical analysis

Statistical Package for the Social Sciences (SPSS) version 26.0 software was employed for statistical processing of general demographic and clinical data, which were represented in the form of mean ± standard deviation. The difference of age and education level between the SCH group and the HC group was compared by two sample t-test, and the difference of gender was compared by chi-square test. Statistical significance was all determined by *p* < 0.05.

The fMRI data were statistically analyzed using the DPABI software package. The sFC and dFC values of bilateral habenula with the whole brain in SCH group and HC group were compared by two sample *t*-test and corrected by gaussian random field (GRF), with age, gender, years of education, and head motion (mean FD) as covariates. The GRF method for multiple comparison correction (voxel-wise *p* < 0.005, cluster-wise *p* < 0.05) was applied.

The sFC and dFC values of statistically significant clusters were generated individually. Then, in the SPSS software, Spearman’s correlation analysis was utilized to explore the correlation of extracted sFC value/dFC value with the PANSS scores by Bonferroni correction (*p* < 0.05).

### 2.8. Validation analysis

Given the small size of habenula, to remove the distortions and signal loss of rs-fMRI signals influenced by the magnetic field inhomogeneity at air-tissue interfaces, we use the Temporal Signal to Noise Ratio (tSNR) based on BRANT (BrainNetome Toolkit)^3^ (30), which is calculated as the average intensity of time series divided by the standard deviation, to generate subject-level whole-brain mask to exclude spurious voxels, then we extracted the tSNR of left and right habenula seeds, and two adjacent but not overlapping cerebrospinal fluid (CSF) seeds (sphere with a radius of 3 mm). Finally, a contrast between the tSNR of bilateral habenula with adjacent CSF seeds was performed.

In order to verify the repeatability of dFC results, in addition to 30 TR, we also performed verification tests on sliding window lengths of 50 and 60 TR according to the suggestion by Liao et al. (29), and the dFC maps for the left and right habenula were reconstructed separately.

## 3. Results

### 3.1. Clinical and demographic characteristics

There were no significant differences in age (*t* = 0.524, *p* = 0.600), gender (χ*^2^* = 0.741, *p* = 0.389), educational level (*t* = 1.964, *p* = 0.050), or head motion (*t* = 1.294, *p* = 0.196) between the SCH and HC group (Table 1).

**TABLE 1:** Demographic and clinical data of SCH and HC groups [Mean (SD)].

	SCH (*n* = 198)	HC (*n* = 199)	*t/*χ^2^	*P*
Age (years)	22.55 ± 8.60	22.17 ± 5.49	0.524	0.600
Sex (male/female)	86/112	95/104	0.741	0.389
Education(years)	10.97 ± 3.12	11.62 ± 3.39	1.964	0.050
Disease duration (months)	6.34 ± 3.70			
**PNASS**				
Positive	20.02 ± 6.54	–		
Negative	21.25 ± 7.23	–		
General	41.13 ± 11.02	–		
Total scores	82.39 ± 21.71	–		
Mean FD (mm)	0.061 ± 0.047	0.067 ± 0.045	1.294	0.196

Sex differences between SCH and HC groups were examined by Chi-square tests. Differences of continuous variables between SCH and HC groups were examined by two sample T-tests. SCH, schizophrenia patients; HC, healthy control; FD, framewise displacement; PNASS, positive and negative syndrome scale; SD, standard deviation.

### 3.2. Differences in static functional connectivity

#### 3.2.1. Left habenula

**FIGURE 1:**
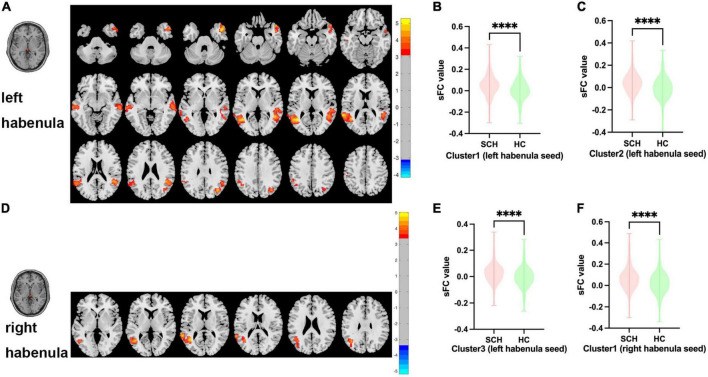
Brain regions showing abnormal sFC values between SCH and HC groups in MNI space using left and right habenula as seeds. **(A)** Significant sFC value differences were observed in cluster1 (left middle temporal gyrus, left superior temporal gyrus), cluster2 (right middle temporal gyrus, right superior temporal gyrus) and cluster3(right temporal pole) using left habenula as seed **(B,C,E)**. Altered sFC values in cluster1, cluster2, and cluster3 between SCH and HC groups using left habenula as seed. **(D)** Significant sFC value differences were observed in cluster1 (left middle temporal gyrus, left superior temporal gyrus, left angular gyrus) using right habenula as seed. **(F)** Altered sFC values in cluster1 between SCH and HC groups using right habenula as seed. ^*⁣*⁣**^Significant at voxel-wise *p* < 0.005, cluster-wise *p* < 0.05 (2-tailed).

Between SCH and HC groups, three clusters exhibit statistical differences of sFC using left habenula as seed, SCH group show increased sFC of left habenula with left middle temporal gyrus, left superior temporal gyrus, right middle temporal gyrus, right superior temporal gyrus, and right temporal pole compared with HC group (see Figure 1 and Table 2).

**TABLE 2:** Whole-brain seed-based static functional connectivity and dynamic functional connectivity analysis results.

ROI	SCH vs HC	Brain areas (L/R)	Cluster size (voxels)	Peak coordinates (MNI)			*T* value
				**x**	**y**	**z**	
**sFC**							
**Habenula _L**	Cluster1(+)	Middle temporal gyrus(L), superior temporal gyrus(L)	633	36,	−72,	33	4.69
	Cluster2(+)	Middle temporal gyrus(R), superior temporal gyrus(R)	620	−39,	−57,	12	5.30
	Cluster3(+)	Temporal pole(R)	218	48,	6,	−30	5.17
**Habenula _R**	Cluster1(+)	Middle temporal gyrus(L), superior temporal gyrus(L), angular gyrus(L)	380	−60,	−45,	15	5.04
**dFC**							
**Habenula_L**	Cluster1(−)	Cuneus gyrus(L/R), Calcarine gyrus(L/R)	134	−3,	−81,	33	−3.71

MNI, Montreal Neurological Institute; ROI, region of interest; R, right; L, left; +, positive; –, negative.

#### 3.2.2. Right habenula

Between SCH and HC groups, one cluster exhibit statistical differences of sFC using right habenula as seed, SCH group show increased sFC of right habenula with left middle temporal gyrus, left superior temporal gyrus, and left angular gyrus compared with HC group (see Figure 1 and Table 2).

### 3.3. Differences in dynamic functional connectivity

#### 3.3.1. Left habenula

**FIGURE 2:**
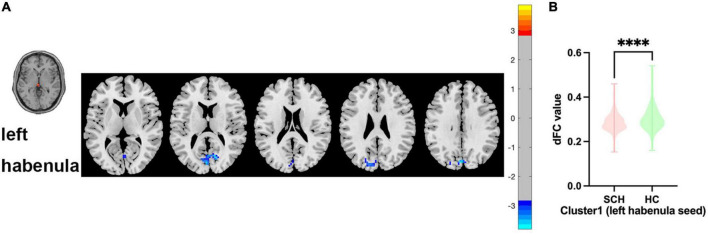
Brain regions showing abnormal dFC values between SCH and HC groups in MNI space using left habenula as seed. **(A)** Significant dFC value differences were observed in cluster1 (bilateral cuneus gyrus, bilateral calcarine gyrus) using left habenula as seed. **(B)** Altered dFC values in cluster1 between SCH and HC groups using left habenula as seed. ****Significant at voxel-wise p < 0.005, cluster-wise p < 0.05 (2-tailed).

Between SCH and HC groups, one cluster exhibit statistical differences of dFC using left habenula as seed, SCH group show decreased dFC of left habenula with bilateral cuneus gyrus and bilateral calcarine gyrus compared with HC group (see Figure 2 and Table 2).

#### 3.3.2. Right habenula

Between SCH and HC groups, no cluster exhibit statistical differences of dFC using right habenula as seed.

### 3.4. Spearman correlational analysis

**FIGURE 3:**
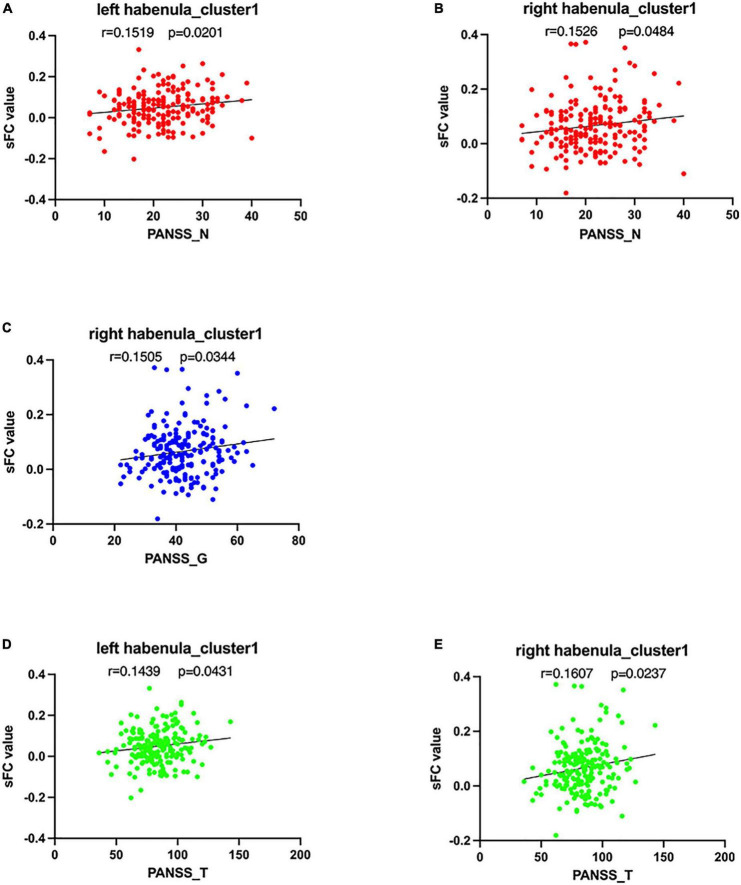
Correlations between abnormal sFC, dFC, values and clinical variables. In schizophrenia patients, **(A)** a positive correlation was observed between the sFC values in the cluster1 using left habenula as seed and PANSS negative sub-scores, **(B)** a positive correlation was observed between the sFC values in the cluster 1 using right habenula as seed and PANSS negative sub-scores, **(C)** a positive correlation was observed between the sFC values in the cluster1 using right habenula as seed and PANSS general sub-scores, and **(D)** a positive correlation was observed between the sFC values in the cluster1 using left habenula as seed and PANSS total scores, **(E)** a positive correlation was observed between the sFC values in the cluster1 using right habenula as seed and P PANSS total scores.

PANSS negative sub-scores were positively correlated with the sFC values of cluster 1 using the left habenula seed in SCH group (*r* = 0.152, *p* = 0.020). PANSS negative sub-scores were positively correlated with the sFC values of cluster 1 using the right habenula seed in SCH group (*r* = 0.153, *p* = 0.048). PANSS general sub-scores were positively correlated with the sFC values of cluster 1 using the right habenula seed in SCH group (*r* = 0.151, *p* = 0.034). PANSS total scores were positively correlated with the sFC values of cluster 1 using the left habenula seed in SCH group (*r* = 0.144, *p* = 0.043). PANSS total scores were positively correlated with the sFC values of cluster 1 using the right habenula seed in SCH group (*r* = 0.161, *p* = 0.024) (see Figure 3). The scores of P2 (hallucination) were negatively correlated with the sFC of cluster 3 using the left habenula seed in SCH group (*r* = –0.226, *p* = 0.019). The scores of G3 (anxiety) were positively correlated with the dFC of cluster 1 using the left habenula seed in SCH group (*r* = 0.212, *p* = 0.008).

### 3.5. Validation results

The mean tSNR of left habenula seed (244.18) and right habenula seeds (321.36) are significantly higher than that of adjacent two CSF seeds (134.26, 135.17) from all subjects, which can rule out the possibility that the habenula signal was physiological to a certain extent (Supplementary materials).

To test the major dFC findings in our study, we applied 2 optional sliding window lengths. The sliding window lengths of 50 and 60 TRs generated findings that were comparable to what we discovered for the 30 TR. The findings of every validation analysis were posted as Supplementary Tables 1, 2.

## 4. Discussion

In our study, we applied the bilateral habenula as seeds to compare the whole brain’s sFC and dFC maps of the first-episode, drug-naïve schizophrenia patients and healthy controls, both left and right habenula were observed increased sFC in SCH group, while left habenula was observed decreased dFC in SCH group. With regard to sFC, the SCH group showed a significant increase in sFC of left habenula with right middle temporal gyrus, right superior temporal gyrus, right temporal pole, left middle temporal gyrus and left superior temporal gyrus compared with HC group; In SCH group, the sFC of right habenula with left middle temporal gyrus, left superior temporal gyrus and left angular gyrus was significantly increased. The dFC of left habenula with bilateral cuneus gyrus and bilateral calcarine gyrus was significantly decreased in SCH group compared with HC group. In addition, our study discovered that the sFC values of statistically significant brain regions with left habenula as seed (right middle temporal gyrus, right superior temporal gyrus) were positively correlated with the negative scores and total scores of PANSS, and sFC values of statistically significant brain regions with right habenula as seed (left middle temporal gyrus, left superior temporal gyrus, left angular gyrus) were positively correlated with the negative scores, general scores and total scores of PANSS. The sFC and dFC values of habenula were negatively correlated with P3 (hallucination) and positively correlated with G2 (anxiety) of PANSS. In conclusion, these results indicate that several brain regions presented abnormal static and dynamic FC with bilateral habenula in schizophrenia patients, including the middle temporal gyrus, superior temporal gyrus, temporal pole, angular gyrus, bilateral cuneus gyrus, and bilateral calcarine gyrus, which act a crucial part in the mechanism of schizophrenia.

Dopamine transmission disorder is an important feature of schizophrenia, and several studies have found that psychiatric symptoms may be driven by hyperdopaminergic state and increased temporal activity in subcortical regions (7, 31). Despite its diminutive size, the habenula was focused by a growing number of psychiatric studies. The habenula mediates the transmission of negative feedback information between dopamine-rich brain regions in the limbic forebrain and dopaminergic neurons in the midbrain structure, as a result of which it can produce a self-rewarding effect, which is thought to act an important part in the pathophysiological process of affective disorders (32, 33). The malfunction of habenula may bring out SCH-like symptoms by mistakenly inhibiting mesencephalon dopaminergic activity (34). Neuroimaging studies have confirmed that the interactions between brain regions in schizophrenia patients have altered (35, 36). Several scholars put forward the theory of “prefrontal-striatum-substantia nigra circuit”, and believed that the damage of this circuit was related to the pathogenesis of mental and cognitive disorder in schizophrenia. The habenula mainly receives information from the “frontal-limbic system-basal ganglia circuit” and the efferent pathway to the midbrain, suggesting that the habenula may be an important hub structure in the forebrain limbic system, striatum and substantia nigra dopamine control system, and a key node connecting the limbic system and mesencephalic dopaminergic neurons, which could have a crucial impact on how schizophrenia develops (32).

Numerous studies have proved that the structural and functional alteration of prefrontal lobe, temporal lobe, basal ganglia, midbrain and other brain regions are related to the occurrence and development of schizophrenia (37), and inseparable fiber and functional connection between the habenula with these regions exists (38). Up to now, only a tiny minority have studied the abnormal FC of habenula in schizophrenia. Zhang et al. reported that the FC of the left habenula with the left medial prefrontal cortex (mPFC), left lingual gyrus and right inferior frontal gyrus (IFG) in patients with SCH has increased, and the FC of the right habenula with the left ventral striatum, caudate and putamen has increased (24). In our study, the SCH group presented several regions which exist abnormal sFC with left and right habenula. The results showed that the sFC of left habenula with bilateral middle temporal gyrus, superior temporal gyrus, and right temporal pole increased, and the sFC of the right habenula with the left middle temporal gyrus, the left superior temporal gyrus, and the left angular gyrus increased, indicating that the habenula may play a role in the pathological changes associated with schizophrenia. Extensive research about schizophrenia have discovered defects in the structure, activation and functional connectivity of the temporal lobe, and several studies have also suggested that temporal lobe dysfunction may be involved in mediating the loss of dopaminergic neuron activity, the enhancement of subcortical dopaminergic nerve conduction and psychosis. Serge et al. reported that the dyslexia of schizophrenia patients is related to the abnormality of dopamine receptors in the middle temporal gyrus, and confirmed that the dopamine receptors in the middle temporal gyrus act an important part in the pathogenesis of cognitive deficits in schizophrenia (39). Mirjam et al. and Monte et al. respectively reported that schizophrenia patients have decreased dopamine D2/D3 receptor binding in the temporal cortex in their PET studies (40, 41). Benedetta et al. study on the resting state network (RSN) of schizophrenia found that the signal transduction of dopamine and 5-HT may be correlated with the FC alteration of RSN such as sensorimotor network (SMN), salience network (SN), and default-mode network (DMN), involving the temporal lobe (42). For the temporal cortex in the auditory system, a mouse model study by Chun et al. found a neuronal mechanism to link the dopamine dysfunction with the generation of auditory hallucinations in schizophrenia (43). Our study also found an increase in functional connectivity of the right habenula with the left angular gyrus. Numerous cognitive processes, including visual communication, visual spatial attention, memory semantics, and social cognition, are mediated by the angular gyrus (44–46). Many previous studies have shown that the angular gyrus, as a part of the default network, showed destroyed structural and functional coupling in schizophrenia patients. Gao et al. utilized methods of rs-FC and granger causality analysis (GCA) to study the internal and causal alteration of habenula in major depressive disorder (MDD) patients after electroconvulsive therapy, and it was found that the functional connectivity between the habenula and the left angular gyrus was increased, and it is concluded that the functional and causal relationship between left angular gyrus and bilateral habenula can be used as a biomarker to distinguish MDD and HC (47). To sum up, we identified aberrant functional connectivity between the habenula and the temporal lobe, angular gyrus in our investigation, which added new evidence for the habenula’s critical role in the neuropathology of schizophrenia. The habenula may affect dopamine information transmission through the altered FC of the habenula with the middle temporal gyrus, the superior temporal gyrus, and the angular gyrus, thus leading to the occurrence of symptoms in schizophrenia patients.

The conventional sFC displays the average connectivity degree between regions, while the dFC shows the potential neuro-endophenotype of schizophrenia, which can better mirror the time-varying nature of internal activity and connectivity, thus revealing the special connectivity patterns that are often lost in sFC analysis. Up to now, a growing number of studies have found that schizophrenia appears dFC alteration in multiple brain regions (48, 49). In this study, the dFC results were different from sFC, and abnormal dFC of significantly abnormal sFC regions with the habenula was absent. In contrast to the HC group, the dFC of the habenula with bilateral cuneus gyrus, calcarine gyrus was significantly reduced in the SCH group, suggesting that the stability of FC of the habenula with the occipital lobe weakened. The cuneus and calcarine gyrus belong to the occipital lobe anatomically, and the occipital lobe is primarily responsible for the prevalence of visual hallucinations in schizophrenia, according to earlier research (50). Li et al. found the altered dFC between the lateral occipital cortex and multiple networks in first-episode schizophrenia patients by using dynamic functional connectivity methods, suggesting that the decreased FC stability of the lateral occipital cortex contributes to the emergence of schizophrenia (51). Bai et al. found that the dopamine receptor-interacting protein in the prefrontal and occipital lobes of schizophrenia were up-regulated, which was related to the abnormal dopamine pathway in schizophrenia (52). In a word, our results may indicate that the FC frequency of left habenula with bilateral cuneus gyrus, calcarine gyrus decreases over time, which may be crucial in the etiology of schizophrenia by affecting the dopamine pathway.

Our study also found that the abnormal sFC of habenula was positively correlated with the negative scores, the general scores and the total scores of PANSS. In terms of symptom scores in PANSS, the sFC and dFC values of habenula were negatively correlated with P3 (hallucinatory) and positively correlated with G2 (anxiety). It is worth mentioning that the correlations of PANSS scores with FC were, despite being significant, rather weak in effect-size. Several studies have found that habenula may play a key role in regulating feedback processing defects of schizophrenia through affecting dopamine pathway (9). Abi-Dargham et al. reviewed several studies and discovered that dopamine dysfunction can help explain the positive symptoms experienced by schizophrenia patients (53). Li et al. observed that decreased activity of the lateral habenula may lead to negative and cognitive symptoms of schizophrenia by downregulating the expression of dopamine receptors in the medial prefrontal cortex in their research on rats (54). Stopper et al. reported that the abnormal dopamine transmission in the habenula may mediate the defects in positive and negative feedback guidance decision-making in schizophrenia patients (55). Our research observed that the decreased sFC between the left habenula and occipital lobe is negatively related to the symptom of hallucination, indicating that the weakened stability of the functional connectivity of the habenula may be the cause of hallucination, while the increased dFC between the left habenula and the left temporal pole which is positively correlated with anxiety indicate that the habenula is strongly linked to the symptom of anxiety in schizophrenia, implying that unstable FC is related to more serious symptom of anxiety. Yet, the absence of this situation of the right habenula may reveal the asymmetry of the habenula’s function, and the abnormal functional connectivity of the left habenula acts a critical role in the occurrence of symptoms in schizophrenia.

There are several limitations in this study that need to be addressed. First of all, we recognize that the habenula is a small structure, and there is a risk of signal leakage from surrounding structures with limited resolution. A more accurate method of segmenting the region is to calculate the exact volume of each subject’s left and right habenula. In the future, a specific habenular nucleus mask based on anatomical manual segmentation and a fMRI of high resolution should be employed. Secondly, this study is a horizontal study of pre-medication schizophrenia patients. In the future, it is necessary to infer whether the abnormal functional connectivity of habenula improves after intervention from the longitudinal study. Third, due to the limitation of imaging resolution, our study did not segment the habenula into medial and lateral part which play an important role in 5-HT and dopamine pathways respectively. In the future, high-resolution imaging is required to separate the medial and lateral habenula, so as to comprehend the respective mechanisms of lateral and medial habenula for mental diseases. Last, we did not evaluate the cognitive function in schizophrenia patients, therefore it is difficult to predict the influence of habenula on the cognitive function of schizophrenia. Therefore, our research is still superficial, in the future, we need to further detailed study about the habenula in schizophrenia, so as to find out the role played by structural and functional abnormalities of habenula in schizophrenia.

## 5. Conclusion

Our study utilized sFC and dFC analysis to examine the aberrant functional connectivity of the habenula with the entire brain regions of first-episode schizophrenia. Additionally, we conduct the correlation analysis of sFC and dFC values with PANSS scores. Our findings revealed that in schizophrenia patients, the sFC between the habenula and the middle temporal gyrus, superior temporal gyrus, temporal pole, angular gyrus is enhanced, while the dFC of the habenula with bilateral cuneus gyrus, calcarine gyrus is weakened. Moreover, the alterations of FC are closly associated with the symptoms of hallucination and anxiety in schizophrenia patients. To sum up, the current research results showed that the habenula of schizophrenia patients presents abnormal static and dynamic functional connectivity with multiple brain regions, which may lead to the symptoms of schizophrenia by affecting the dopamine pathway and provide a new neural mechanism.

## Data availability statement

The original contributions presented in this study are included in this article/Supplementary material, further inquiries can be directed to the corresponding author.

## Ethics statement

The studies involving human participants were reviewed and approved by Ethics Committee of The First Affiliated Hospital of Zhengzhou University. Written informed consent to participate in this study was provided by the participants’ legal guardian/next of kin.

## Author contributions

KX contributed to the conception and design of this study and wrote the first draft of the manuscript. JChen and YW recruited the participants and performed the MRI examination. KX, SH, and CW performed data processing. YC performed the statistical analysis. YZ, XS, and JCheng provided critical revision of the manuscript. All authors contributed to the article and approved the submitted manuscript.

## Footnotes

1
http://rfmri.org/dpabi

2
https://github.com/junqianxulab/Habenula_fMRI_ROIs

3
https://sphinx-doc-brant.readthedocs.io/en/latest/index.html
