# Supplementary material for: Altered static and dynamic functional connectivity of habenula in first-episode, drug-naïve schizophrenia patients, and their association with symptoms including hallucination and anxiety
Source: Front Psychiatry. 2023 Jan 19;14:1078779. doi: 10.3389/fpsyt.2023.1078779 (PMC9892902; doi:10.3389/fpsyt.2023.1078779)
Supplement: Supplementary file 1 [file Data_Sheet_1.docx]

Supplementary Material

## **
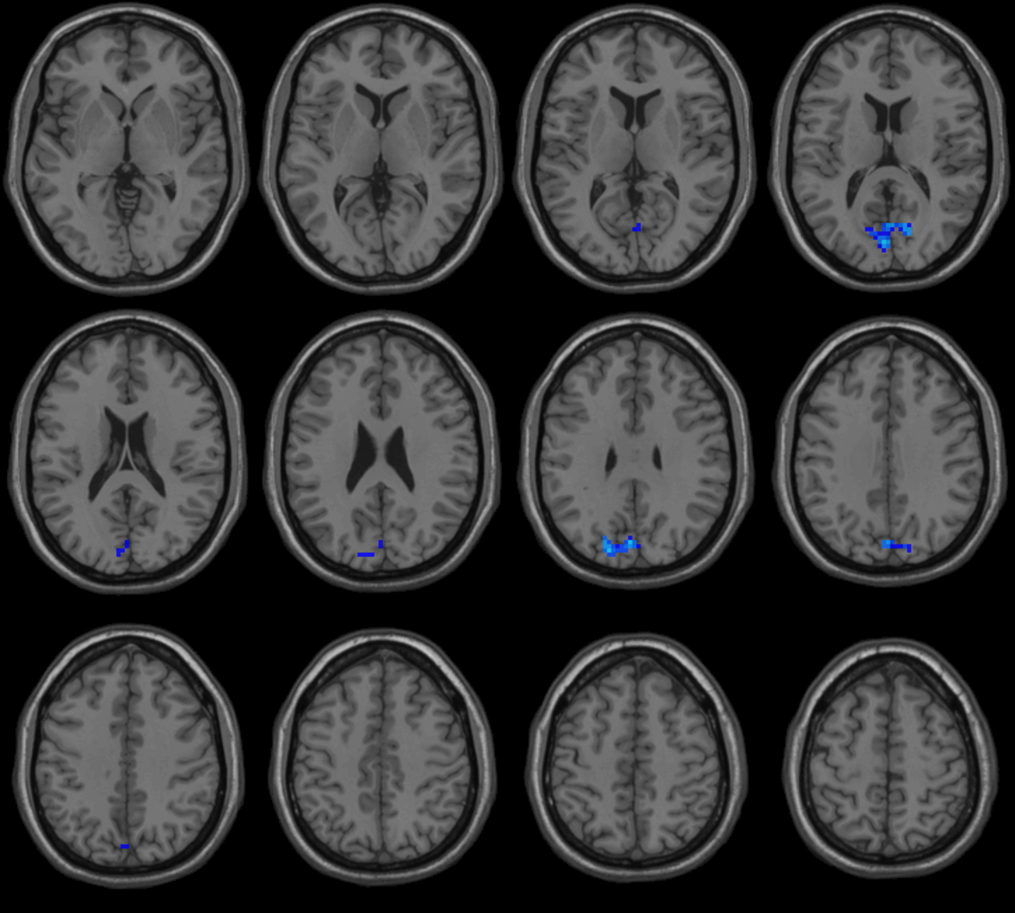
**Supplementary Figures

**Supplementary Figure 1.** Brain regions showing abnormal dFC values between SCH and HC groups in MNI space using left habenula as seed with the sliding window length of 50 TR. Significant dFC value differences were observed in cluster1(bilateral calcarine gyrus, bilateral cuneus gyrus, left superior occipital gyrus) using left habenula as seed.


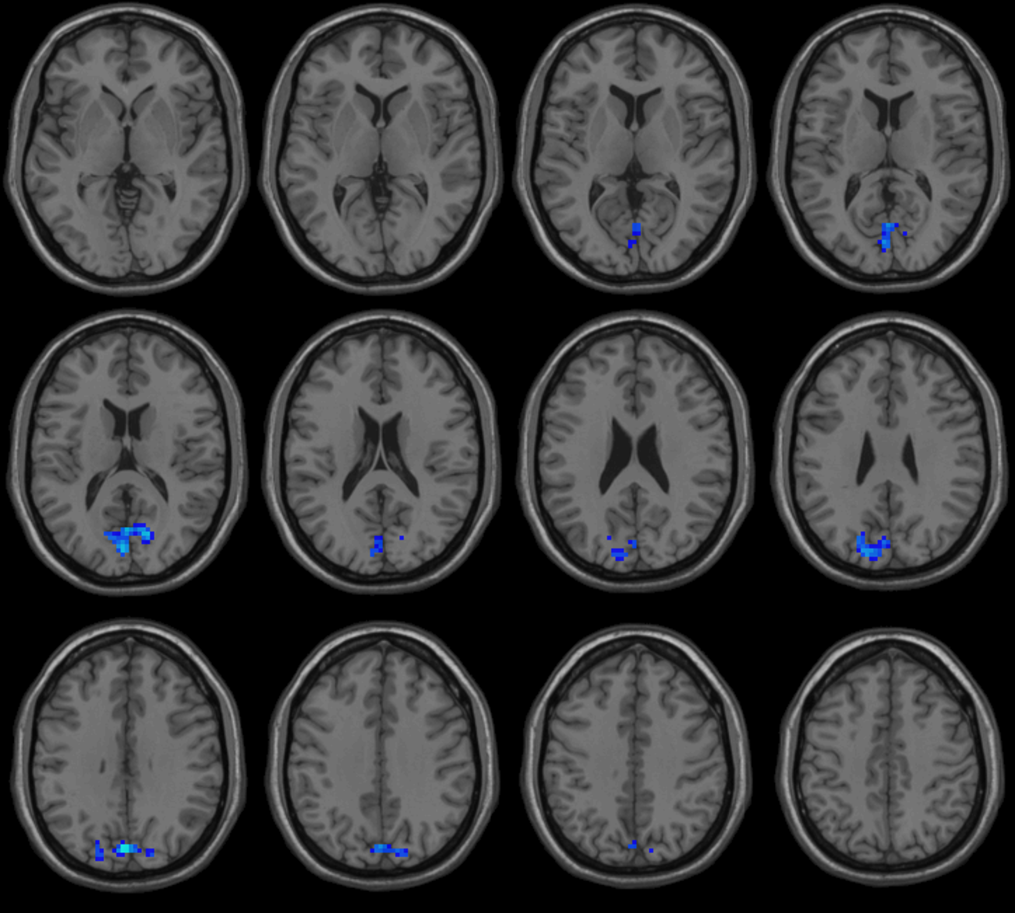


**Supplementary Figure 2.** Brain regions showing abnormal dFC values between SCH and HC groups in MNI space using left habenula as seed with the sliding window length of 60 TR. Significant dFC value differences were observed in cluster1(bilateral calcarine gyrus, bilateral cuneus gyrus, left superior occipital gyrus, left superior occipital gyrus, lef precuneus gyrus) using left habenula as seed.
